# Comparison between 10 and 12 mg doses of intrathecal hyperbaric (0.5%) bupivacaine on sensory block level after first spinal failure in cesarean section: A double-blind, randomized clinical trial

**DOI:** 10.3389/fmed.2022.937963

**Published:** 2022-10-04

**Authors:** Nahid Manouchehrian, Farshid Rahimi-Bashar, Azar Pirdehghan, Fatemeh Shahmoradi

**Affiliations:** ^1^Department of Anesthesiology, Fatemi Medical Center, Hamadan University of Medical Sciences, Hamadan, Iran; ^2^Anesthesia and Critical Care Department, Hamadan University of Medical Sciences, Hamadan, Iran; ^3^School of Public Health and Research Center for Health Sciences, Hamadan University of Medical Sciences, Hamadan, Iran; ^4^Faculty of Medical Sciences, Hamadan University of Medical Sciences, Hamedan, Iran

**Keywords:** motor block, cesarean section, bupivacaine, failed spinal, spinal anesthesia, sensory block

## Abstract

**Background:**

Reducing adverse effects during cesarean delivery and improving the quality of sensory blocks with appropriate doses of intrathecal hyperbaric bupivacaine can play an important role in the safe management of cesarean delivery. The aim of this study was to compare the doses of 10 and 12 mg of intrathecal hyperbaric bupivacaine 0.5% on sensory block level after first spinal failure in cesarean section (CS).

**Methods:**

In this double-blind, randomized clinical trial, 40 candidates of CS after first spinal failure with class I-II based on American Society of Anesthesiologists (ASA) were randomly assigned into two equal groups (*n* = 20). Group A and B received the spinal anesthesia with 10 mg and 12 mg of hyperbaric bupivacaine (0.5%), respectively. Maximum levels of sensory block, motor block quality, and vital signs were measured in two groups by 60 min after SPA. Incidence of SPA complications during surgery were also recorded. Data were analyzed by SPSS ver.21 software using repeated measures analysis of variance at 95% confidence interval (CI) level.

**Results:**

Excellent quality of sensory blocks and complete quality of motor blocks were achieved in all participants (100%). However, the mean time to onset of anesthesia (4.47 ± 0.69 vs. 3.38 ± 0.47, *P* < 0.001) and time to reach T10 level (60.73 ± 11.92 vs. 79.00 ± 19.21, *P* < 0.001) in the Group A, were significantly shorter than in the patients of Group B. The incidence of hypotension (*P* = 0.001), nausea/vomiting (*P* = 0.007) and bradycardia (*P* = 0.012) as well as administration of ephedrine and atropine were significantly higher in Group B compared to Group A.

**Conclusion:**

Spinal anesthesia can be safely repeated with a 10 mg of hyperbaric bupivacaine 0.5% in a caesarean section after the initial spinal failure.

**Clinical trial registration:**

[https://en.irct.ir/trial/40714], identifier [IRCT20120915010841N20].

## Introduction

Spinal anesthesia (SPA) is the most common, safest, and most rational choice for cesarean section (1). SPA is secure and effective, but not a 100% successful technique and complications have been part of the method (2), including failed or insufficient sensory block (3), postdural puncture headache (PDPH) (4), hypotension (5), bradycardia (6), nerve damage (7), nausea and vomiting (8). A specific level of sensory block is required in any surgery performed under SPA. In cesarean section (CS), the level of T4-T6 anesthesia is appropriate (9). Elevated level of sensory block (≥ T4) will cause hypotension, nausea and vomiting, decreased level of consciousness and maternal discomfort. Conversely, lower level of sensory block (≤ T6) will not provide adequate anesthesia for CS, and causes discomfort and dissatisfaction in the patient (10). Intrathecal anesthetic spread has an unpredictable extent and duration that can be related to various factors such as dosage, patient variables, cerebrospinal fluid (CFS) volume, injection rate, and injection site (11).

One of the issues of SPA is the failure of spinal anesthesia, which means that SPA has been performed but not enough sensory block has been provided for surgery (12). Failed SPA can be identified as partial or incomplete spinal block within 10 minutes after hyperbaric bupivacaine injection and 25 minutes after isobaric bupivacaine anesthesia (13). Failure rates in SPA have been reported from 1 to 17% in various 1-17% (14). However, major studies have reported a prevalence range of 2 to 4% (12, 15). Obesity, dry cerebrospinal fluid (CSF), bloody CSF, improper dose, incorrect anesthesia distribution, multiple lumbar puncture attempts, use of the L4/L5 interspace, history of previous anesthesia and technical errors are significantly associated with failed SPA (3, 16). Complete failure of the SPA can be managed by switching to general anesthesia or by repeating the SPA procedure (12). However, since most pregnant patients are at risk for aspiration and intubation problems, general anesthesia carries a relatively higher risk for this population and re-performing SPA is a better and safer choice (17).

Administration of an appropriate dose hyperbaric bupivacaine can minimize potential side effects while improving block quality (18). However, the optimal intrathecal dose of hyperbaric bupivacaine for SPA is still being debated. Previous studies have investigated and demonstrated the effects of different doses on sensory and motor blocks in different ways (19, 20). In addition, very few studies have been found on the appropriate dose of hyperbaric bupivacaine for cesarean section during repeated spinal anesthesia (21). Therefore, we conducted this study to compare the doses of 10 and 12 mg of intrathecal hyperbaric (0.5%) bupivacaine on sensory block level and PDPH after first spinal failure in cesarean section.

## Materials and methods

### Study design

This prospective, double blind, parallel-group, randomized clinical trial study was conducted in Fatemiyeh Hospital in Hamadan, Iran, from July 2018 to July 2019. The protocol study was reviewed and approved by the Ethics Committees of Hamadan University of Medical Sciences (IR.UMSHA.REC.1398.232). This study Registered at Iranian Registry of Clinical Trials (IRCT20120915010841N20). Written informed consent were obtained from each patient. The study was conducted in accordance with the Declaration of Helsinki of the World Medical Association (22). This study was performed and reported in accordance with the recommendations of the Consolidated Standards of Reporting Trials (CONSORT) statement (23).

### Population of study and sample size

The study population consisted of parturient with class I-II of American Society of Anesthesiologists (ASA), aged 18 to 46 years, the height range 170-155 cm, a second SPA candidate after the first failed SPA (Bromage score 0 and no sensory block even at L4 dermatome after 10 min of first hyperbaric bupivacaine injection). Patients with a history of hypertensive pregnancy disorders, heart disease, Bromage scale >0, lack of pinprick sensation below umbilicus after spinal anesthesia, and of allergies to the study drug were excluded from the trial.

### Randomization and blinding

Forty parturient were selected by a convenience sampling method based on inclusion criteria. Patients were then randomized into two SPA groups containing 10 mg of hyperbaric bupivacaine 0.5% (Group A) and 12 mg of hyperbaric bupivacaine 0.5% (Group B). Equal number of patients were assigned to each group using the block randomization method (*n* = 20). Patients were assigned to Group A or Group B on a computer-generated random number selected by the patient using Random Allocation Software © (RAS; Informer Technologies, Inc., Madrid, Spain). The level of spinal block and the duration of hemodynamic sensory variables were compared between the two groups. Both the patients and the evaluator were blind to the assignments.

### Pre-SPA procedure

The Pre-SPA procedure was started for each patient as follows: Lactated Ringer serum (10 ml/kg) was injected using an 18-gauge needle depending on the patient’s weight. Standard monitoring includes electrocardiography, pulse oximetry and non-invasive blood pressure (NIBP), vital signs such as systolic blood pressure (SBP), diastolic blood pressure (DBP), mean blood pressure (MBP), heart rate (HR) and oxygen saturation (SpO2) was measured and recorded using an X162 monitor (Saadat Company, Iran).

### First SPA procedure

SPA contains 10 mg of hyperbaric bupivacaine 0.5% (AstraZeneca Company, France) plus 2.5 μg of sufentanil (Abu Rayhan Company, Iran) using Quinke needle size 25 through lower lumbar (L3-4 or L4-5) intervertebral spaces in a sitting position was administered. The patient immediately lay on his back with a wedge under his right hip and was monitored for vital sign (SBP, DBP, MBP, HR, and SpO2). The effects of sensory and motor blocks were observed within 5 min, those that did not show efficacy within 5 min were observed for an additional 5 min and tested for motor and sensory blocks again. Those ASA I-II patients with insufficient motor and sensory block (Bromage score 0 and no sensory block even at L4 dermatome after 10 min of first hyperbaric bupivacaine injection) were considered for inclusion in the present study and they randomly (as described above) allocated to either Group A (10 mg of hyperbaric bupivacaine 0.5%) or Group B A (12 mg of hyperbaric bupivacaine 0.5%).

### Repeat procedure of SPA

Patients in Group A received 10 mg and patients in Group B received 12 mg of 0.5% high hyperbaric bupivacaine (AstraZeneca Company, France), respectively and 2.5 μg of sufentanil (Abu Rayhan Company, Iran). Taking the same precautions, a 25-gauge Quincke needle was placed above or below the gap in the first attempt and the submucosal block was performed again (one space above in patients where previous spinal was attempted at L4-5 interspace or one space below in those patients who had previous spinal at L3-4 interspace) by a senior anesthesiologist who have worked in anesthesiology for over 10 years. The patient immediately lay on his back, a wedge under his right buttock displaced the left uterus, and monitoring (sensory block, motor block, and vital signs) was initiated. Surgery could be started after the initiation of spinal anesthesia was confirmed by a proper movement block of the lower extremities without a pinching sensation. If the patient complained of a pin-stab sensation 10 minutes after repeated spinal cord administration, general anesthesia was given and the patient was excluded from the study.

### Measurements

The maximum level of sensory block was assessed by the pin-prick method using a 25-gauge needle, the time to reach the maximum level of anesthesia and the time to reach the T10 level as primary outcomes, were recorded for each patient. The sensory block quality and pain were assessed using the visual analog scale (VAS). VAS scores were recorded by creating a handwritten mark on a 10 cm line indicating the chain between “excellent” and “poor” (24), which described excellent postoperative quality as none (0), mild (< 3), moderate (3-6), or severe (7-10). The quality of the motion block was assessed using the Bromage Scale (25). A modified Bromage Scale was used: 0 = no motor block; 1 = able to flex knee free movement of feet, unable to raise extended leg (partial motor block); 2 = free movement of feet only (almost complete motor block); 3 = unable to move hips, knees, feet (complete motor block). Sedation was evaluated by Ramsay scale, it divides a patient’s level of sedation into six categories ranging from severe agitation to deep coma; 1 = anxious and agitated or restless or both; 2 = co-operative, oriented and tranquil; 3 = responding to commands only; 4 = brisk response to light glabellar tap or loud auditory stimulus; 5 = sluggish response to light glabellar tap or loud auditory stimulus; 6 = no response to stimulus (26).

Vitals parameters including SBP, DBP, MBP, HR, and SpO2 were measured at baseline (pre-SPA procedure), immediately after SPA procedure, and 2,4,6,8, 10, 15, 20, 30, 40, 50, and 60 min of post postoperatively. SBP less than 90 mmHg and bradycardia (heart rate less than 60 beats per minute) were treated with incremental intravenous doses of 10 mg ephedrine and 0.5 mg intravenous atropine, respectively. Finally, the amount of ephedrine and atropine used, the occurrence of nausea and vomiting during surgery, the time of onset of anesthesia and the maximum level of anesthesia (using a needle or pinprick), the quality of sensory (VAS scores) and motor block (Bromage Scale), the time of anesthesia to T10, sedation score (Ramsay scale) and Apgar score (in minutes 1 and 5) for infants was examined and recorded for each participants. Nausea and Vomiting, headache, hypotension (BP < 90/60mmHg), bradycardia (HR < 60/min), chills and high spinal were recorded during procedure for each patient. In addition, one week after surgery, patients were evaluated and questioned by researcher over the phone about the presence or absence of postdural puncture headache (PDPH).

### Statistical analysis

Power calculations was done based on primary outcome, time to reach T10 level in two group of study (60.73 ± 11.92 in 10 mg of hyperbaric 0.5% vs. 79.00 ± 19.21 in 12 mg of hyperbaric 0.5%, *P* < 0.001). Analyses according to the sample size of 20 patients in each group with considering the type I error (α) set as two-sided 5% (Z1-α/2 = 1.96) and type II error (β) set as 20% (Z1-β = 0.84), estimated the power of the test equal to 100%. It should be noted that with a power level of 80 and 95% confidence interval (CI), a sample size equal to 13 patients in each group was sufficient to detect clinically significant differences between the two groups. Analyzing the power of the test using Stata 11 software. Variables were expressed as mean ± standard deviation (SD) or percentage (%) for continuous and discrete variables, respectively. Results were analyzed by independent *t*-test (between groups), and paired *t*-test (within group) for parametric data and Mann–Whitney *U*-test for non-parametric data. Fisher’s exact test and Chi-square test were used for categorical data as appropriate. The Shapiro-Wilk test was conducted to test whether the data were normally distributed. Using a general linear model, hemodynamic changes and complications between the two groups were compared using a repeated measurement ANOVA test, with the baseline values (age) used as covariates in the model. The assumption of sphericity was addressed by Mauchly’s test of sphericity, and when the assumption was not satisfied, the Greenhouse-Geiser correction of *P*-value were utilized. To assess the effect of intervention, the analysis of covariance (ANCOVA) was used after controlling for baseline measures and confounders in a two-step hierarchical model. Logistic regression analysis was used to predict incidence of complications according to influencing 12 mg dose of hyperbaric bupivacaine 0.5% compare to 10 mg, and the significant variables were reported as odds ratio (OR) with 95% confidence interval (CI). GraphPad Prism 9© (GraphPad Software Inc., La Jolla, CA) was used to show the changes of hemodynamic parameters in two groups of study (12 mg vs. 10 mg of hyperbaric 0.5%) over times. Statistical analysis was carried out using SPSS software (ver.21) (SPSS Inc., IL, Chicago, United States). In all analyses, *P*-values less than 0.05 were considered as significant.

## Results

### Participants of study

**FIGURE 1:**
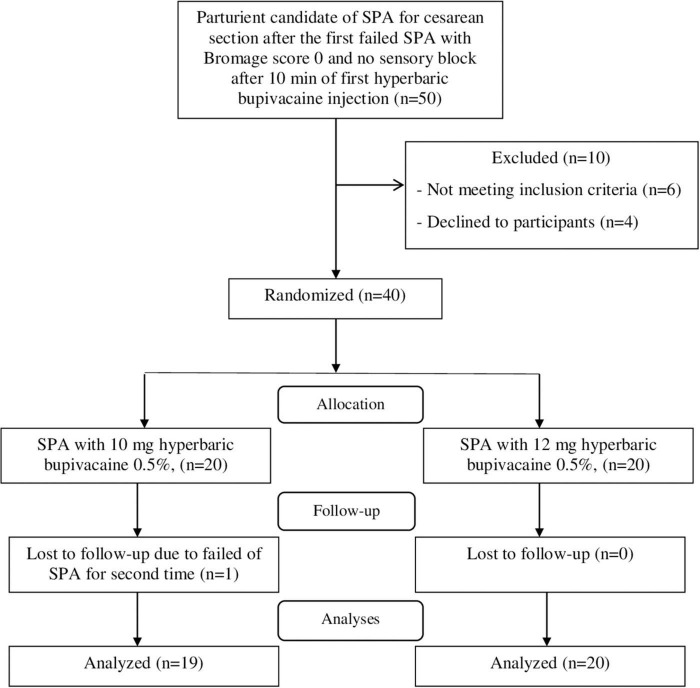
CONSORT flow diagram.

A total of forty parturient were included in the study and Figure 1 shows the patient registration flow chart. Fifty parturient with class I-II of ASA, who candidate of SPA for non-emergent cesarean section after the first failed SPA with Bromage score 0 and no sensory block after 10 min of first hyperbaric bupivacaine injection were screened for eligibility criteria. Out of 50 cases, 40 patients met the inclusion criteria and randomly assigned into two equal groups (*n* = 20); Group A (received 10 mg of hyperbaric bupivacaine 0.5%) and Group B (received 12 mg of hyperbaric bupivacaine 0.5%). During the intervention and follow-up stages, only one patient in the group A underwent general anesthesia due to failed of SPA and was excluded from the study. Totally 39 patients were analyzed, 19 and 20 patients in the Group A and B, respectively.

### Demographic and baseline hemodynamic parameters

Comparison of baseline demographics and hemodynamic parameters between the two groups of study is presented in Table 1. There were no statistically significant differences in demographic and baseline hemodynamic parameters of parturient such as; age (*P* = 0.722), SBP (*P* = 0.985), DBP (*P* = 0.398), MBP (*P* = 0.531), HR (*P* = 0.372) and SpO2 (*P* = 0.682) in the Group A and Group B.

**TABLE 1:** Comparison of demographic and baseline hemodynamic parameters between the two study groups.

Variables		Group A (*n* = 19)	Group B (*n* = 20)	*P*-value	95% Confidence interval (CI)
Age	Mean ± SD	30.94 ± 4.81	31.70 ± 7.96	0.722	−3.54 to 5.05
	(Range)	(24-42)	(18-44)		
Systolic BP	Mean ± SD	121.53 ± 11.48	121.60 ± 13.19	0.985	−7.97 to 8.11
	(Range)	(97-136)	(100-150)		
Diastolic BP	Mean ± SD	71.84 ± 11.31	75.20 ± 13.08	0.398	−4.59 to 11.31
	(Range)	(47-97)	(52-99)		
MBP	Mean ± SD	86.63 ± 12.29	89.35 ± 14.37	0.531	−5.98 to 11.41
	(Range)	(61-111)	(59-119)		
HR	Mean ± SD	87.94 ± 17.27	92.55 ± 14.48	0.372	−5.71 to 14.92
	(Range)	(56-120)	(62-118)		
SpO2	Mean ± SD	96.84 ± 1.06	97.00 ± 1.29	0.682	−0.615 to 0.931
	(Range)	(95-99)	(93-99)		

Group A (who received 10 mg of hyperbaric bupivacaine 0.5%), Group B (who received 12 mg of hyperbaric bupivacaine 0.5%), HR: Heart Rate, BP: Blood Pressure, MBP: Mean Blood Pressure, SpO2: oxygen saturation.

### Spinal anesthesia characteristics and outcomes

Comparison of spinal anesthesia characteristics and outcomes between the two groups of study are presented in Table 2. Excellent and complete quality of sensory and motor blocks was observed in all participants in the both groups. However, the mean time to onset of anesthesia (4.47 ± 0.69 vs. 3.38 ± 0.47, *P* < 0.001) and the mean time to reach T10 level (60.73 ± 11.92 vs. 79.00 ± 19.21, *P* < 0.001) in the Group A were significantly shorter than in the Group B. According to the results, in most patients in Group A the sensory level reached to T6 (n = 17, 89.5%), while the sensory level of more than half of the patients in Group B reached to T4 (n = 13, 65%). In terms of sensory level at recovery, nearly half of the patients (47.4%) in Group A had sensory level T12, while 50% of participants in the Group B had sensory level T8. There was statistically significant difference between two groups of the study in terms on maximum sensory level (*P* < 0.001) and also in sensory level in recovery (*P* < 0.001). The use of Ephedrine (85% vs. 31.6%, *P* < 0.001) and Atropine (30% vs. 0) in the group B who received SPA with 12 mg of hyperbaric bupivacaine 0.5% was significantly higher than the Group A. And finally, the satisfaction of patients in the Group B was significantly lower than the Group A. However, there was no significant difference in the Apgar scores of the neonates in the first minute (8.78 ± 0.71 vs. 8.95 ± 0.39, *P* = 0.387), and fifth minutes (9.94 ± 0.22 vs. 9.90 ± 0.31, *P* = 0.591) between two groups.

**TABLE 2:** Comparison of spinal anesthesia characteristics and outcomes between the two study groups.

Variables		Group A (*n* = 19)	Group B (*n* = 20)	*P*-value
Time to reach T10 level	Mean ± SD (min)	60.73 ± 11.92	79.00 ± 19.21	< 0.001*
Maximum sensory level	T2 T4 T6	0 2 (10.5) 17 (89.5)	3 (15) 13 (65) 4 (20)	< 0.001*
Time to onset of anesthesia	Mean ± SD (min)	4.47 ± 0.69	3.38 ± 0.47	< 0.001*
Sensory block quality	Excellent (%)	19 (100)	20 (100)	–
	Moderate (%)	0	0	
	Poor (%)	0	0	
Motor block quality	Complete (%)	19 (100)	20 (100)	–
	Semi-complete (%)	0	0	
	Non-motion block (%)	0	0	
Sensory level in recovery	T8	2 (10.5)	10 (50)	< 0.001*
	T10	8 (42.1)	9 (45)	
	T12	9 (47.4)	1 (5)	
Ephedrine doses consumed	Median (IQR)	0 (0-10)	20 (20-30)	< 0.001*
Ephedrine consumed	Yes (%)	6 (31.6)	17 (85)	< 0.001*
Atropine consumed	Yes (%)	0	6 (30)	0.009*
Apgar score	Mean ± SD (1min)	8.78 ± 0.71	8.95 ± 0.39	0.387
	Mean ± SD (5 min)	9.94 ± 0.22	9.90 ± 0.31	0.591
Satisfaction rate	Low (%)	3 (15.8)	13 (65)	0.003*
	Moderate (%)	2 (10.5)	1 (5)	
	High (%)	10 (52.6)	5 (25)	
	Very high (%)	4 (21.1)	1 (5)	

Group A (who received 10 mg of hyperbaric bupivacaine 0.5%), Group B (who received 12 mg of hyperbaric bupivacaine 0.5%), * statistical significant.

### Changes in hemodynamic parameters and sedation rate over time

**FIGURE 2:**
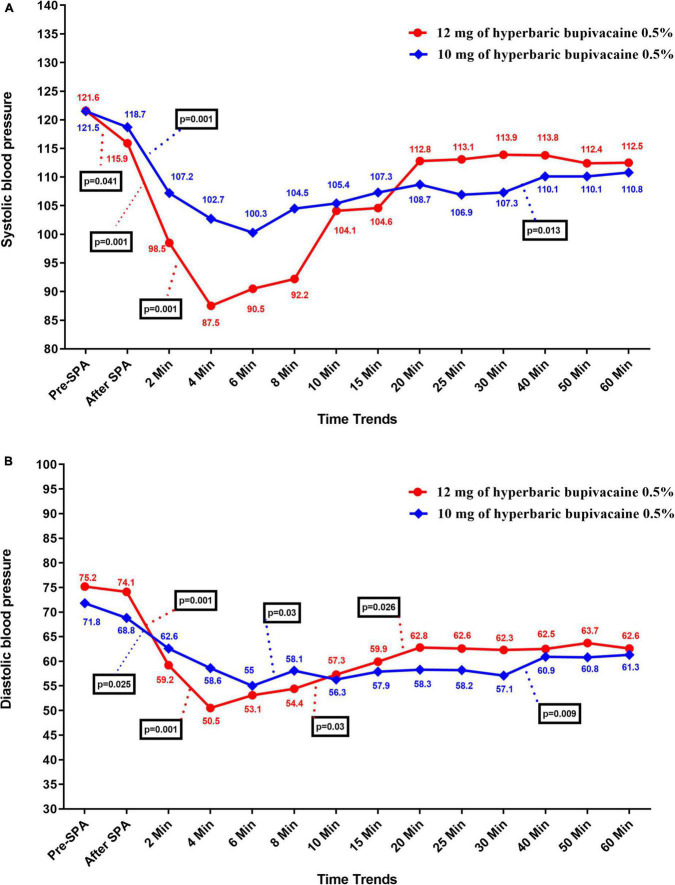
Changes **(A)** systolic and **(B)** diastolic blood pressure in two groups of study over times, *P-values shows statistically significant between two times within groups.

The time trend of hemodynamic parameters (SBP, DBP, MBP, HR, and SpO2) in the two study groups is presented in Table 3. This parameters were recorded at pre-SPA and immediately after SPA and then every 2 min up to 10-min, and then every 5 min up to 30-min, and then every 10 min up to 60-min after injection of anesthetic drug. Figure 2A shows the mean values of SBP changes in each group over time. The results showed that there was no significant difference in SBP between the two groups except for 4 and 8 min when SBP in Group A was significantly higher than Group B (*P* = 0.002 and *P* = 0.038, respectively), also in 30 min that the SBP in Group B was higher than Group A (*P* = 0.033). In within group, the effect of time on SBP in each group was statistically significant (a within-subject difference based on time effect) (*P* = 0.05). However, based on repeated measures analysis of variance (RMANOVA), the trend of changes in SBP levels between the two groups was not statistically significant (group * time interaction or an interaction effect) (*P* = 0.362).

**TABLE 3:** Comparison of hemodynamic parameters and sedation rate based on Ramsay scale in two groups of study.

Variables/Groups		Pre- SPA	After SPA	2 Min	4 Min	6 Min	8 Min	10 Min	15 Min	20 Min	25 Min	30 Min	40 Min	50 Min	60 Min	P-value ##	P-value ###	P-value ####
SBP	**A**	121.52 (11.48)	118.73 (11.51)	107.2 (13.9)	102.7 (11.8)	100.3 (16.4)	104.5 (15.4)	105.42 (9.61)	107.31 (10.05)	108.7 (11.8)	106.94 (11.57)	107.3 (10.1)	110.2 (9.17)	110.1 (8.60)	110.84 (9.05)	**0.002**	**< 0.001**	0.362
	**B**	121.60 (13.19)	115.95 (12.35)	98.5 (15.4)	87.5 (16.1)	90.5 (19.4)	92.2 (19.9)	104.15 (15.48)	104.65 (23.87)	112.8 (9.16)	113.1 (8.40)	113.9 (8.58)	113.8 (5.10)	112.4 (5.26)	112.5 (4.41)	**0.011**		
P-value#		0.985	0.471	0.072	**0.002**	0.097	**0.038**	0.761	0.655	0.243	0.069	**0.033**	0.131	0.298	0.469			
DBP	**A**	71.84 (11.3)	68.89 (13.9)	62.63 (10.6)	58.63 (13.2)	55 (11.3)	58.05 (10.8)	56.31 (7.68)	57.94 (8.42)	58.36 (8.96)	58.21 (8.83)	57.15 (8.43)	60.94 (7.43)	60.84 (7.04)	61.36 (6.72)	**0.002**	**< 0.001**	0.643
	**B**	75.20 (13.1)	74.05 (12.8)	59.2 (12.5)	50.55 (9.92)	53.10 (14.2)	54.4 (13.1)	57.3 (10.3)	59.9 (8.57)	62.85 (7.84)	62.6 (7.80)	62.3 (7.80)	62.55 (7.13)	63.75 (7.09)	62.6 (5.33)	**< 0.001**		
P-value#		0.398	0.236	0.365	**0.037**	0.649	0.325	0.739	0.478	0.105	0.108	0.053	0.496	0.207	0.513			
MBP	**A**	86.63 (14.3)	84.52 (13.3)	75.57 (11.1)	72.63 (12.9)	69.78 (12.1)	72.84 (12.2)	71.31 (8.01)	73.73 (8.89)	73.42 (9.44)	72.84 (8.68)	75.42 (9.91)	75.47 (8.77)	77.21 (6.53)	77.26 (6.81)	**0.004**	**< 0.001**	0.973
	**B**	89.35 (14.3)	86.85 (12.2)	70.75 (12.9)	61.75 (12.7)	65.10 (15.3)	66.30 (14.7)	75.12 (12.1)	75.45 (8.28)	78.55 (8.04)	78.45 (8.00)	78.60 (5.88)	78.95 (5.88)	78.80 (4.49)	78.65 (3.36)	**0.005**		
P-value#		0.531	0.573	0.220	**0.012**	0.299	0.142	0.801	0.537	0.075	**0.043**	0.267	0.153	0.380	0.422			
HR	**A**	87.94 (17.2)	93.47 (15.7)	97.5 (18.3)	102.9 (16.1)	97.94 (13.6)	97.26 (11.4)	95.89 (12.5)	94.84 (12.4)	94.94 (10.6)	94.36 (10.3)	93.89 (9.97)	94.05 (7.26)	93.94 (8.48)	92.73 (7.15)	0.296	0.126	0.067
	**B**	92.55 (14.4)	99.40 (28.3)	101.1 (22.3)	93.7 (29.2)	98.10 (20.8)	104.6 (15.3)	106.7 (11.9)	105.2 (10.6)	105.5 (9.19)	104.1 (6.64)	99.90 (7.90)	99.15 (6.50)	95.60 (5.26)	94.80 (4.68)	0.454		
P-value#		0.372	0.428	0.595	0.232	0.979	0.101	**0.009**	**0.008**	**0.002**	**0.001**	**0.044**	**0.026**	0.467	0.291			
SpO_2_	**A**	96.84 (1.06)	97.42 (1.12)	97.73 (0.99)	97.73 (0.99)	97.47 (1.71)	97.78 (1.27)	97.84 (1.16)	97.63 (1.57)	97.78 (1.18)	97.94 (1.12)	97.94 (1.12)	97.42 (2.06)	97.94 (1.12)	97.47 (2.06)	0.259	0.107	**0.036**
	**B**	97 (1.29)	97.35 (0.87)	96.90 (1.41)	96.65 (1.42)	96.55 (1.39)	96.75 (1.11)	96.80 (1.01)	97 (1.02)	97.10 (0.91)	97.15 (0.98)	97.25 (0.85)	97.25 (0.85)	97.20 (0.89)	97.25 (0.85)	0.287		
P-value#		0.632	0.826	**0.040**	**0.009**	0.072	**0.010**	**0.005**	0.144	**0.048**	**0.024**	**0.038**	0.735	**0.027**	0.658			
Sedation rate	**A**	–	2	2	1.93 (0.25)	1.87 (0.34)	1.93 (0.25)	2.18 (0.40)	2.25 (0.44)	2.25 (0.44)	2.17 (0.39)	2.25 (0.44)	2.18 (0.40)	2	2	–	0.057	0.102
	**B**	–	2	1.95 (0.39)	1.75 (0.55)	1.70 (0.57)	1.95 (0.39)	2.05 (0.22)	2.05 (0.22)	2.10 (0.30)	2	2.05 (0.22)	2.05 (0.22)	2.05 (0.22)	2	–		
P-value#		–	–	0.616	0.216	0.288	0.913	0.203	0.089	0.242	0.052	0.089	0.203	0.379	–			

Group A: 10 mg of hyperbaric bupivacaine 0.5%, Group B: 12 mg of hyperbaric bupivacaine 0.5%, SBP: systolic blood pressure, DBP: diastolic blood pressure, MAP: Mean Arterial Pressure, HR: Heart Rate, SpO2: oxygen saturation, *P* < 0.05 was considered as significant, # *P*-value based on independent t-test and analysis of covariance (ANCOVA) adjusted for age between two groups, ##*P*-value based on paired t-test within group, ### Time main effect based on two way analysis of variance with repeated measures (RMANOVA), #### Assessing the interaction effect of group and time based on RMANOVA after Greenhouse-Geiser correction (adjusted and non-adjusted models). Bold values are *P* < 0.05 which was considered as significant.

As shown in Figure 2B, there was no significant difference in DBP between the two groups except at 4 min, when DBP in Group A was significantly higher than Group B (58.63 ± 13.25 vs. 50.55 ± 9.92, *P* = 0.037). Within the group, the effect of time on DBP was statistically significant in each group (difference within the subject based on the effect of time) (*P* < 0.05). However, the trend of changes in DBP levels between the two groups (group * time interaction or an interaction effect) (*P* = 0.643).

**FIGURE 3:**
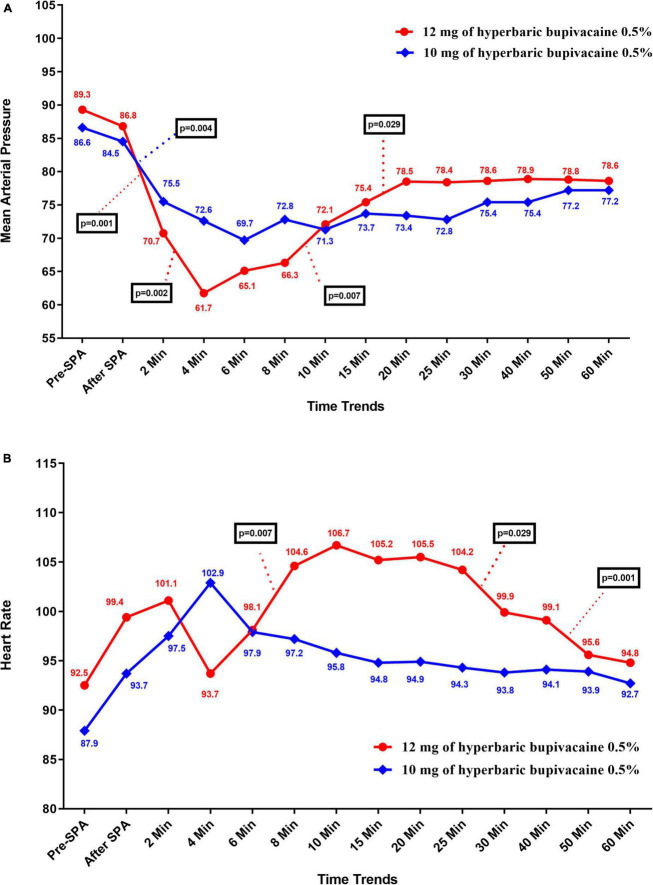
Changes **(A)** main blood pressure and **(B)** heart rate in two groups of study over times, * *P*-values shows statistically significant between two times within groups.

Figure 3A shows the mean values for changes of MBP in each group over times. According to the results, there was no significant difference in MBP between the two groups except at 4 min (72.63 ± 12.95 vs. 61.75 ± 12.73, *P* = 0.012) and 25 min (72.84 ± 8.68 vs. 78.45 ± 8, *P* = 0.043) when MBP in Group A was significantly higher and lower than Group B, respectively. In within group, the effect of time on MBP was statistically significant in Group A (*P* = 0.004) and Group B (*P* = 0.005) (a within-subject difference based on time effect). However, the trend in changes in MBP levels was not statistically significant between two groups (group * time interaction or an interaction effect) (*P* = 0.935).

Figure 3B shows the mean values for changes of HR in each group over times. The results showed that there was a significant difference in HR between the two groups at 10 min to 40 min, when HR in Group B was significantly higher than Group A (*P* < 0.05). In within group, time effect on HR was not statistically significant in Group A (*P* = 0.296) and Group B (*P* = 0.454) (a within-subject difference based on time effect). Moreover, the trend in changes in HR levels was not statistically significant between two groups (group × * time interaction or an interaction effect) (*P* = 0.067).

**FIGURE 4:**
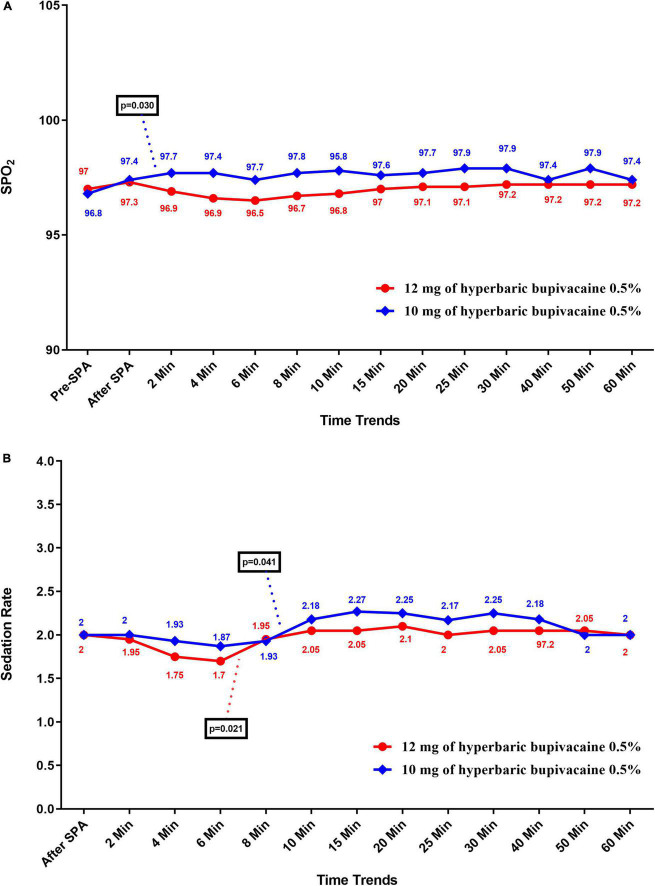
Changes **(A)** SpO2, and **(B)** sedation rate in two groups of study over times, * *P*-values shows statistically significant between two times within groups.

Figures 4A,B shows the mean values for changes of SpO2 and sedation rate in each group over times, respectively. According to our findings, the mean SpO2 was significantly higher in the Group A than in the Group B (*P* < 0.05), except at pre-SPA (*P* = 0.682), immediately after SPA (*P* = 0.826), at 6 min (*P* = 0.072), 15 min (*P* = 0.144) and 60 min (*P* = 0.658). In within group, time effect on SpO2 was not statistically significant in each group (a within-subject difference based on time effect) (*P* > 0.05). While, the trend in changes in SpO2 levels was statistically significant between two groups (group × time interaction or an interaction effect) (*P* = 0.036). In terms of sedation rate, no significant difference was observed between the two groups and also within each group (*P* > 0.05). In addition, the trend in changes in sedation rate was not statistically significant between two groups (group * time interaction or an interaction effect) (*P* = 0.102).

### Complications

Table 4 shows comparison of complications related to the SPA procedure during operation, at recovery and after operation in two groups of study. According to our findings, hypotension was a common SPA side effect in both groups, which was occurring in 59% of the all participants. The results indicated that the incidence of hypotension (85 vs. 31.6%, *P* = 0.001), nausea/vomiting (70 vs. 26.3%, *P* = 0.007) and bradycardia (30% vs. 0, *P* = 0.012) were significantly higher in Group B compared to Group A. However, there was no significant difference in chills, headache, pain, high spinal and PDPH in the two groups (*P* > 0.05). Based on logistic regression analysis, 12 mg of hyperbaric bupivacaine (0.5%) can be increases the risk of hypotension (OR: 12.278, 95% CI: 2.573-58.589, *P* = 0.002), nausea/vomiting during operation (OR: 6.533, 95% CI: 1.613-26.469, *P* = 0.009) and ephedrine consumed (OR: 12.278, 95% CI: 2.573-58.589, *P* = 0.002) (Table 5).

**TABLE 4:** Comparison of SPA-related complications in two groups of study.

Side effects	Group A (*n* = 19)	Group B (*n* = 20)	*P*-value
**During operation**			
Nausea and Vomiting Headache Hypotension Bradycardia Chills High spinal	5 (26.3%) 0 6 (31.6%) 0 6 (31.6%) 0	14 (70%) 1 (5%) 17 (85%) 6 (30%) 6 (30%) 3 (15%)	0.007* 0.513 0.001* 0.012* 0.915 0.125
**At recovery**			
Nausea and Vomiting Pain	0 0	1 (5%) 1 (5%)	0.513 0.513
**After operation**			
PDPH	1 (5.3%)	1 (5%)	0.744

Group A: 10 mg of hyperbaric bupivacaine 0.5%, Group B: 12 mg of hyperbaric bupivacaine 0.5%, *, *P* < 0.05 was considered as significant, PDPH: Postdural puncture headache.

**TABLE 5:** Logistic regression analysis of influencing 12 mg dose of hyperbaric bupivacaine 0.5%, to predict incidence of complications.

Variables	Logistic regression analysis	
	OR (95% CI)	*P*-value
Nausea and Vomiting during operation (yes vs. no)	6.533 (1.613-26.469)	0.009*
Headache during operation (yes vs. no)	0.972 (0.879-1.074)	0.571
Headache after operation (yes vs. no)	0.947 (0.055-16.309)	0.970
Hypotension (yes vs. no)	12.278 (2.573-58.589)	0.002*
Chills (yes vs. no)	0.929 (0.238-3.619)	0.915
Ephedrine consumed (yes vs. no)	12.278 (2.573-58.589)	0.002*

*P < 0.05 was considered as significant.

## Discussion

The failure of a SPA to produce adequate block is not an uncommon occurrence in cesarean section. However, little information is available to provide guidance on duplicate dosing. The main purpose of this clinical trial was to compare the doses of 10 mg and 12 mg of intrathecal hyperbaric bupivacaine (0.5%) on sensory block level after first spinal failure in cesarean section. The excellent quality of sensory block and the complete quality of motor block were obtained in all participants. Although, both doses (10 mg and 12 mg) of intrathecal hyperbaric bupivacaine (0.5%) showed similar satisfactory block profiles. But our results revealed that the SPA with 12 mg hyperbaric bupivacaine (0.5%) can increase the mean anesthesia time and time to reach the T10 level.

Technical errors are common causes of failed spinal such as drug deposition at lower spinal level than surgical site, improper rate of injection, failure to detect dural puncture, needle from inside/outside the dural sac, patient co-operation, needle in ventral epidural space, and cerebrospinal fluid (CSF) tap. Therefore, due to the risk of aspiration and intubation problems in pregnant patients, repeating the procedure of SPA is the safer option (3, 27). Managing failure SPA or repeating the procedure is an event that is of concern to both the patient and the anesthesiologist, and several factors must be considered. Adequate dose of local anesthetic and the skills of anesthetist to prevent technical errors are of this factors. The superior quality of sensory blocks and the complete quality of motor blocks in both research groups may be attributed to the ability of anesthesia providers to prove their effectiveness with experienced hands (28). SPA is safer in skilled hands, but several factors are believed to affect SPA, including anatomical abnormalities such as kyphoscoliosis, sclerosis, and spinal stenosis following previous intrathecal surgery or chemotherapy and reduced anesthetic potency due to prolonged exposure to light (12, 29, 30).

In terms of dose of local anesthetic, previous studies have suggested that a dose of 12 mg (2.4 ml) of bupivacaine provides reliable anesthesia for cesarean section (27, 31). However, in the repetition of the SPA procedure there is a fear of over-expansion of the sensory block (12, 32). As in this study, high spinal block was occurred in 3 patients who received 12 mg hyperbaric bupivacaine (0.5%). So, our findings showed that the lower dose (10 mg) of anesthetic drug is safer and did not observed any high spinal block in Group A. On the other hand, by reviewing the literature, we found an association between hypotension and bradycardia in cesarean section with a higher dose of anesthetic, which was completely consistent with the results of this study (21, 33, 34). Our findings indicated that the higher dose of bupivacaine (12 mg) was related to higher nausea/vomiting, hypotension, and bradycardia as well as administration of more ephedrine and atropine, which ultimately reduces patient satisfaction. The high and very high satisfaction rate of parturients in this study was significantly higher in Group A compare to Group B (73.7 vs.30%, *P* < 0.05). Evidence suggests that overall satisfaction level of parturients decreases with number of attempt, pain during block, inadequate intraoperative analgesia, postoperative nausea/vomiting, hypotension, bradycardia and headache during operation and high level of PDPH (35). However, by ensuring the quality of spinal anesthesia, improving the clinical skills of anesthesiologists, preventing side effects, and educating mothers about cesarean section under local anesthesia and familiarity with the process, patients You can increase your satisfaction (36). Therefore, choosing the adequate dose of anesthetic drug can be increases the quality of local anesthesia and prevents side effects, and subsequently increase patient satisfaction. The results of this study are consistent with previous studies showing that reducing the dose of local anesthesia during repeated spinal anesthesia is safe and satisfactory (21, 32).

The limitations of our study were, we compared the only two doses of bupivacaine, based on the known optimal doses and low sample size. However, a large sample size study s need to be conducted to determine the optimal dose of hyperbaric bupivacaine that can be safely and successfully used to repeat SPA in parturient women. However, the important teaching concepts of this study are as follows; considering that failure in spinal anesthesia often happens to assistants and less experienced anesthesiologists, and the text books do not mention reducing the dose of bupivacaine in spinal re-injection, if these specialists regardless of reducing the dose of spinal drug, use bupivacaine with the same initial dose as mentioned in the text books, it can lead to an increase in the spinal level and cause problems for patients and anesthesiologists. Since we also work in the obstetric anesthesia training department and deal with spinal failure, we decided to investigate the reduction of bupivacaine dose following spinal failure so that less experienced specialists can use this experience and have fewer problems such as increasing the level of block and hypotension, etc.

## Conclusion

Spinal anesthesia can be safely repeated with a 10 mg of hyperbaric bupivacaine 0.5% in a caesarean section after the initial spinal failure. SPA with 10 mg of hyperbaric bupivacaine 0.5% can be improves the quality of local anesthesia, prevents side effects and, as a result, increases patient satisfaction.

## Data availability statement

The raw data supporting the conclusions of this article will be made available by the authors, without undue reservation.

## Ethics statement

The studies involving human participants were reviewed and approved by Ethics Committees of Hamadan University of Medical Sciences (IR.UMSHA.REC.1398.232). The patients/participants provided their written informed consent to participate in this study.

## Author contributions

FR-B, NM, and AP: study concept and design. FR-B, NM, and FS: analysis and interpretation of data, study concept and design, and Critical revision of the manuscript for important intellectual content. AP: acquisition of data and drafting of the manuscript. FS: statistical analysis. All authors contributed to the article and approved the submitted version.
